# Retraction: Transforming growth factor-β1 promotes M1 alveolar macrophage polarization in acute lung injury by up-regulating DNMT1 to mediate the microRNA-124/PELI1/IRF5 axis

**DOI:** 10.3389/fcimb.2025.1705838

**Published:** 2025-09-19

**Authors:** Frontiers Editorial Office

The journal retracts the August 24, 2021 article cited above.

Following publication, concerns were raised regarding the integrity of the images in the published figures. The authors failed to provide a satisfactory explanation during the investigation, which was conducted in accordance with Frontiers’ policies.

This retraction was approved by the Chief Editors of Frontiers in Cellular and Infection Microbiology and the Chief Executive Editor of Frontiers. The authors received a communication regarding the retraction and had a chance to respond. This communication has been recorded by the publisher.

